# In Vitro and In Vivo Translational Insights into the Intraoperative Use of Antiseptics and Lavage Solutions Against Microorganisms Causing Orthopedic Infections

**DOI:** 10.3390/ijms252312720

**Published:** 2024-11-26

**Authors:** Bartłomiej Dudek, Malwina Brożyna, Michał Karoluk, Mariusz Frankiewicz, Paweł Migdał, Konrad Szustakiewicz, Tomasz Matys, Adrian Wiater, Adam Junka

**Affiliations:** 1 “P.U.M.A.”, Platform for Unique Model Application, Department of Pharmacy, Wroclaw Medical University, Borowska 211, 50-534 Wroclaw, Poland; malwina.brozyna@umw.edu.pl; 2 Faculty of Mechanical Engineering, Department of Laser Technologies, Automation and Production Organization, Wrocław University of Science and Technology, Ignacego Łukasiewicza 5, 50-371 Wroclaw, Poland; michal.karoluk@pwr.edu.pl (M.K.); mariusz.frankiewicz@pwr.edu.pl (M.F.); 3 Department of Bees Breeding, Institute of Animal Husbandry and Breeding, Faculty of Biology and Animal Science, Wroclaw University of Environmental and Life Sciences, Chełmońskiego 38C, 51-630 Wroclaw, Poland; pawel.migdal@upwr.edu.pl; 4 Department of Polymer Engineering and Technology, Faculty of Chemistry, Wroclaw University of Science and Technology, Wyb. Wyspianskiego 27, 50-370 Wroclaw, Poland; konrad.szustakiewicz@pwr.edu.pl; 5 The Department and Clinic of Angiology and Internal Medicine, Wroclaw Medical University, Borowska 213, 50-556 Wroclaw, Poland; tomasz.matys@umw.edu.pl; 6 Department of Industrial and Environmental Microbiology, Institute of Biological Sciences, Faculty of Biology and Biotechnology, Maria Curie-Skłodowska University, Akademicka 19, 20-033 Lublin, Poland; adrian.wiater@mail.umcs.pl

**Keywords:** intraoperative use, orthopedic surgery, antiseptics, lavage solutions, biofilm, *Galleria mellonella* larvae

## Abstract

The growing antibiotic resistance of microorganisms causing postoperative infections following orthopedic surgeries underscores the urgent need for localized antiseptic and lavage delivery systems to enhance infection control. This study evaluates the in vitro effectiveness of antiseptic and lavage solutions—including polyhexanide, povidone–iodine, low-concentrated hypochlorite, Ringer’s solution, and saline—against *Staphylococcus epidermidis*, *Staphylococcus aureus* MRSA, *Cutibacterium acnes*, *Corynebacterium amycolatum*, *Pseudomonas aeruginosa*, and *Candida albicans*. Using microplate models (Minimum Inhibitory Concentration, Minimum Biofilm Eradication Concentration, and Biofilm-Oriented Antiseptic Test assays), flow-based models (Bioflux system), and surfaces relevant to orthopedic implants (e.g., stainless steel disks/screws, Co-Cr-Mo, Ti-Al-Nb orthopedic alloys, and ultra-high-molecular-weight polyethylene), as well as a bio-nano-cellulose scaffold representing tissue, we assessed the solutions’ activity. The cytotoxicity of the solutions was evaluated using osteoblast and keratinocyte cell lines, with additional in vivo insights gained through the *Galleria mellonella* larval model. The results show that polyhexanide-based solutions outperformed povidone–iodine in biofilm eradication in most tests applied, particularly on complex surfaces, whereas iodine demonstrated higher cytotoxicity in applied in vitro and in vivo tests. Low-concentration hypochlorite solutions exhibited minimal antibiofilm activity but also showed no cytotoxicity in cell line and *G. mellonella* larval models. These findings highlight the importance of careful antiseptic selection and rinsing protocols to balance infection control efficacy with tissue compatibility in orthopedic applications.

## 1. Introduction

The Review on Antimicrobial Resistance estimated that AMR could result in 10 million deaths annually by 2050, and this projection played a pivotal role in highlighting AMR as one of the most critical health challenges of the 21st century. However, despite this increased global attention, the inconsistent implementation and funding of national action plans have resulted in variable progress, leaving the burden of AMR as an ongoing concern [1].

Orthopedic postoperative infections are complications that occur after surgical procedures involving bones, joints, or associated soft tissues. The microbial causative agents are often the patient’s own microbiota or microbes that reach the surgical site by contaminated instruments or through the environment [2]. Orthopedic infections and their treatment are particularly problematic when biomaterials like implants or prosthetics are involved, as they provide surfaces for microbial adhesion and biofilm formation. A significant portion of orthopedic infections are caused by skin-associated microbes entering the surgical site, such as *Staphylococcus epidermidis* and *S. aureus* (including methicillin-resistant *S. aureus*, MRSA), both known for their biofilm-forming capabilities on orthopedic implants. Additionally, *Cutibacterium acnes* and *Corynebacterium amycolatum*, which are part of the patient’s microbiota, can cause persistent infections (especially those localized in the spine area) after surgery. The Gram-negative *Pseudomonas aeruginosa* or the fungus *Candida albicans* are less frequently implicated; nevertheless, they also can be isolated as etiological factors of immunocompromised orthopedic patients or when biomaterials are involved [3,4,5]. 

Orthopedic-related infections can lead to biofilm formation, implant failure, chronic complications, and the need for revision surgery, posing a significant threat to therapeutic success. Despite advances in surgical techniques and perioperative care, the incidence of surgical site infections (SSIs) in orthopedic procedures remains a significant issue, with rates ranging from 0.5 to 2.5%, depending on the type of surgery and patient risk factors [6].

Antibiotic prophylaxis remains one of the key strategies in preventing these infections, with standard guidelines recommending the administration of antibiotics within 60 min prior to the incision to ensure optimal tissue concentration during surgery. Commonly, first-generation cephalosporins such as cefazolin are employed to target pathogens like *S. aureus* and *S. epidermidis* [7].

Systemic antibiotic therapy is commonly employed to prevent the dissemination of microorganisms throughout the patient’s body. However, many antibiotics face challenges in penetrating the mineralized structure of bones and, to an even greater extent, the metallic alloys of orthopedic implants. Consequently, there is an increasing emphasis on the use of locally acting agents, such as antiseptics and lavaseptics, to address these limitations.

Moreover, the growing emergence of antibiotic-resistant organisms, including methicillin-resistant MRSA and multidrug-resistant Gram-negative bacteria, is increasingly limiting the efficacy of conventional prophylactic regimens. The presence of implants and biomaterials’ non-organic surfaces also provides an optimal environment for biofilm formation, which further complicates treatment by reducing antibiotic penetration into these microbial communities [8,9]. As a result, despite the use of systemic antibiotics, infections can persist, leading to implant failure and the need for revision surgery. These challenges highlight the urgent need for alternative strategies, such as localized antiseptic delivery systems and novel antimicrobial approaches, to improve infection control [10].

Antiseptics are chemical agents designed to kill microorganisms on living tissues, particularly to fight or to prevent the spread of infection. They possess a direct, broad spectrum of antimicrobial activity, targeting bacteria, fungi, and certain types of viruses [11]. Antiseptic agents like povidone–iodine or polyhexanide are applied to treat chronic wound infections, while alcohol-based products are used to disinfect the skin before surgical procedures. In contrast, lavage solutions, also referred to as lavaseptics, are primarily used to mechanically flush out debris, blood, and contaminants (including microbial biofilm) from wounds or cavities during procedures or treatment. While lavaseptics may or may not contain antimicrobial agents, their main function is to clean the area through mechanical means (with the use of appropriate surfactant agents) rather than directly kill microorganisms [12].

However, there is some overlap between these two types of agents, as antiseptics can be used in the character of lavage solutions in surgical settings, providing both antimicrobial activity and mechanical cleansing thanks to surfactants added to their composition. Additionally, some lavaseptics are formulated with antimicrobial properties, blurring the distinction between antiseptics and lavaseptics. This mixed situation makes it essential to clearly define the intended use of each solution—whether for its antimicrobial effects, mechanical flushing, or a combination of both—depending on the surgical context.

The use of antiseptics and lavaseptics in managing chronic wounds is a well-established procedure, with antiseptics providing both antimicrobial protection and mechanical cleansing to promote healing [12].

However, in orthopedic surgery, infection prevention and control present a more complex challenge. The balance between achieving effective antimicrobial activity and avoiding potential harm to the patient’s tissues and cells is critical. Antiseptics, while effective at reducing the microbial load, also cause cytotoxicity, damaging healthy tissues and potentially impairing healing, particularly when used around delicate tissues, such as in bone and joint surgeries [13]. This creates an ongoing debate about the optimal use of antiseptics in orthopedic surgery. One key question is how quickly antiseptics should be rinsed out after application, as residual antiseptic could continue to interact with tissue postoperatively, potentially causing harm. Lavage solutions without antimicrobial properties may be employed to flush out antiseptics, but the exact protocol for balancing efficacy against tissue safety remains a subject of ongoing research and clinical consideration in orthopedic procedures.

Therefore, the aim of the presented investigation is to provide additional data from in vitro studies on microbial biofilms performed on surfaces relevant to orthopedic context (metals, their alloys, soft biological scaffolds) and treated with antiseptics containing polyhexanide, povidone–iodine, and hypochlorite, as well as lavaseptics (Ringer’s solution, saline). Understanding that the balance between antimicrobial efficacy and the potential harm to patient tissues is essential for improving outcomes, we also conducted a parallel line of investigation and explored the effects of antiseptic and lavage agents displayed both on human cells in vitro and on a living model organism (*Galleria mellonella* larvae) in vivo. In no way do we seek, by means of this publication, to provide conclusive results—these must be verified through further experimental and clinical studies. However, this is a critically important area of research, given the rising concerns over infection control and antibiotic resistance, especially in the context of implant-related surgeries. Therefore, our objective was to identify potentially promising directions for further research contributing to the ongoing discussion of antiseptic and lavaseptic use in the prevention of infections related to orthopedic surgeries.

## 2. Results

In the first line of investigation, a routine MIC and MBEC assessment, in 96-well plates and with 24 h exposure time, of the analyzed products and microorganisms (n = 25 for each species) was performed. The results, presented in the form of median MBC and MBEC, are shown in Table 1, while particular MICs and MBECs towards particular strains are given in Supplementary Material S1. Gram-positive cocci (*S. epidermidis* and *S. aureus*) were the most sensitive to PHMB and LS; both these products contain polyhexanide as an active substance, but with different concentrations (PHMB contains 2.5 times more polyhexanide than LS). The cocci were not sensitive to low-concentration hypochlorite in the applied range of concentrations (with the highest concentration of 50%). Not surprisingly, the cocci (and other tested microorganisms) were also not sensitive to liquids that do not contain antimicrobial components (R, NaCl).

The Gram-negative *P. aeruginosa* displayed a higher tolerance to PHMB than Gram-positive cocci, with median MBC and MBEC equal to 3.13% and 25%, respectively. The MBEC values of *C. acnes* and *C. amycolatum* were also higher than those recorded in cocci, yet still within the range of the concentrations applied. The iodine-containing product (B) also displayed activity towards planktonic and biofilm-forming cells in the microtitrate plate setting; nevertheless, the MBC/MBEC values recorded were higher (less favorable) than what was observed for PHMB-containing liquids. In any case, the values of MBEC were lower than the value of MIC; the highest difference between these two values (32 times) was observed for *C. acnes*.

In the next experimental setting, referred to as the Biofilm-Oriented Antiseptic Test (B.O.A.T.), shorter (and more clinically relevant) exposures to antiseptic/lavaseptic liquids were applied (Table 2). Moreover, the B.O.A.T. test also allows for the measurement of the activity of a working solution (undiluted product, i.e., 100%), in contrast to the routine microtitrate plate model (Table 1), where a maximum of 50% of solutions can be tested. The data provided by this version of B.O.A.T. are of a qualitative nature, i.e., they indicate whether the complete eradication of a biofilm of a specific strain after exposure to a specific liquid did or did not occur (i.e., it provides a yes/no type of result).

As shown in Table 2, liquids that do not contain antimicrobial components (NaCl, R) did not display any antibiofilm activity in the B.O.A.T. test, similarly to the low-concentration hypochlorite product (G) and lower-concentration polyhexanide product (LS). The PHMB and B liquids did not manifest antibiofilm activity towards all *S. epidermidis*, *S. aureus*, and *C. acnes* strains in the shortest time applied of 30’. Overall, the B product acted more efficiently in the 30’ exposure time and completely eradicated the biofilm formed by 60% of *C. amycolatum* strains, while no *C. amycolatum* biofilm-forming strains were completely eradicated by the PHMB liquid. In 1 h of exposure, the PHMB liquid was more effective towards *C. albicans* than the B liquid; in turn, both the PHMB and B antiseptics were equally effective against *P. aeruginosa biofilms*.

The data presented in Table 1 and Table 2 were obtained using static conditions for biofilm development, where the polystyrene bottom of a 96-well plate served as the surface of biofilm growth. In the subsequent line of investigation, a porous, elastic, and mesh-like polymeric surface made of bacterial cellulose was applied to mimic the surface of soft tissue.

**Figure 1:**
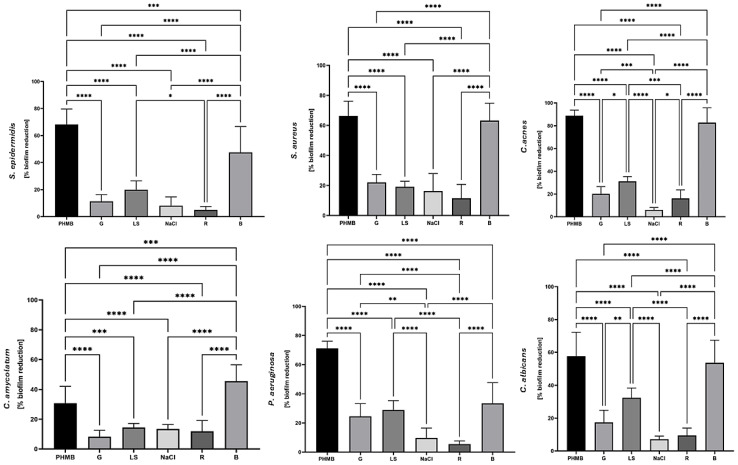
The [%] reduction in biofilm formed on a Bacterial Cellulose Model after exposure to PHMB, G, LS, NaCl, R, and B liquids. Mann–Whitney test with Brown–Forsythe test; differences were considered statistically significant when *p* value > 0.0001. NaCl: saline; PHMB: 0.1% polyhexanide + poloxamer surfactant; G: low-concentration hypochlorite; LS: 0.04% polyhexanide + poloxamer surfactant; R: Ringer’s solution; B: iodine-containing antiseptic. Asterisks indicate the strength of statistical significance.

The data presented in Figure 1 indicate that the G, LS, NaCl, and R products displayed a low (~20%) ability to eradicate pathogenic biofilms, regardless of the species they were applied against. The NaCl and R products contain no antimicrobial compounds; therefore, the observed drop in biofilm biomass was likely caused by the subsequent rinsing procedures performed within the test protocol, i.e., by a mechanical disruptive force. Similar levels of eradication were observed when the G and LS products were applied. The average level of biofilm reduction, regardless of the species applied, were ~65% and~50% for the PHMB or iodine-containing B product, respectively. All differences between the reduction caused by PHMB and B were statistically significant (*p* < 0.0001). The data presented in Figure 1 are cohesive with those in Table 1 and Table 2 in this regard, in that the PHMB and B products displayed significantly higher antibiofilm activity than G and LS and—unsurprisingly—than NaCl and R. In turn, the results obtained by means of the Bacterial Cellulose Model indicate that PHMB acted significantly stronger (*p* < 0.0001) than the B Liquid against pathogenic biofilms of *S. epidermidis*, *S. aureus*, *C. acnes*, *C. amycolatum*, and *P. aeruginosa*, but not *C. albicans*, in contrast to the results obtained by means of the B.O.A.T. test (Table 2), where the B liquid performed better than PHMB overall.

**Figure 2:**
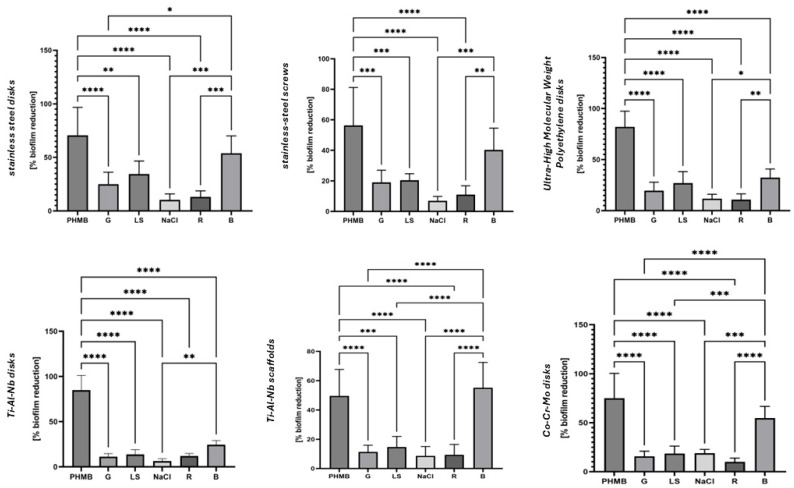
[%] of biofilm reduction, regardless of species, measured in flow conditions. The asterisks indicate the statistical significance of biofilm reduction; Mann–Whitney test with Brown–Forsythe test; differences were considered statistically significant when *p* value > 0.0001. NaCl: saline; PHMB: 0.1% polyhexanide + poloxamer surfactant; G: low-concentration hypochlorite; LS: 0.04% polyhexanide + macrogol surfactant; R: Ringer’s solution; B: iodine-containing antiseptic. Asterisks indicate the strength of statistical significance.

In the fourth experimental model, biofilms grown on abiotic surfaces commonly used in orthopedic implants were rinsed for a clinically relevant duration of 5 min. The data presented in Figure 2 indicate that the application of a rinsing force translates, to some extent, into biofilm removal, even when liquids devoid of antimicrobial component (R, NaCl) are applied. Interestingly, in the case of liquids containing low concentrations of antimicrobials (G, LS), the level of eradication was higher than the one observed for R and NaCl when stainless steel, UHMWP, and Ti-Al-Nb disks (but not Ti-Al-Nb scaffolds) were used as surfaces for biofilm growth, contrary to the results obtained in the Bacterial Cellulose Model (Figure 1), where all these products displayed a similar level of activity. Nevertheless, the level of biofilm eradication did not exceed 35% and 25%, respectively, when LS or G were applied.

Another important observation was that the more complex the surface (mesh, screws), the lower the eradication observed, compared to the smooth surfaces of disks. Out of the two antiseptics (PHMB and B), recognized as the most effective in the earlier-presented datasets (Table 1 and Table 2, Figure 1), PHMB manifested significantly higher antibiofilm activity (*p* < 0.0001) when stainless steel disks and screws, UHMW polyethylene disks, Ti-Al-Nb disks, and Co-Cr-Mo disks—but not Ti-Al-Nb scaffolds—served as the surface for biofilm growth.

**Figure 3:**
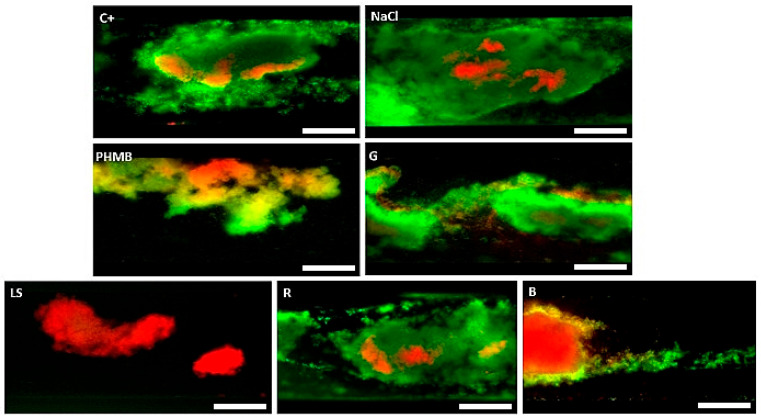
Biofilm subjected to rinsing with different liquids. C+: water; NaCl: saline; PHMB: 0.1% polyhexanide + poloxamer surfactant; G: low-concentration hypochlorite; LS: 0.04% polyhexanide + macrogol surfactant; R: Ringer’s solution; B: iodine-containing antiseptic. The white bar represents a length of 50 µm. Red/orange color—dead/damaged biofilm-forming cells; green color—live/non-damaged cells.

The next investigation also covered flow conditions; however, this time, it was performed for biofilms formed by Gram-positive *S. aureus* only (Figure 3). This experimental line included strictly controlled microfluidic conditions and fluorescent microscopic technique in which live (intact), and dead (damaged) biofilm-forming cells are distinguished by green or red/orange color, respectively.

The obtained results indicate no visible differences, understood as live/dead cell ratio and overall amount of biofilm biomass, after treatment with water, or NaCl, or R solution. Interestingly, low-concentration hypochlorite (G liquid) applied in these microfluidic conditions was able to visibly diminish the area covered with biofilm. Nevertheless, this reduction was weaker compared to the biofilms exposed to PHMB, LS, and B. Regarding these three liquids, LS, although containing a lower concentration of polyhexanide than PHMB, exhibited a higher ability to remove the biofilm than the latter antiseptic. In turn, the B antiseptic, although able to efficiently kill the bacteria, was at the same time unable to efficiently push biofilm out of the microcapillaries. This phenomenon was seen as a clogged bulk of dead (red-dyed) cells in the capillaries (Figure 3, picture “B”).

This relatively higher efficacy of LS than PHMB in microcapillary conditions may be partially explained by the performed measurements of wettability, where LS exhibited different properties than other liquids applied (Supplementary Material S2). It was found that for hydrophobic surfaces (PLLA with water contact angle of ~85°, PLLA-HAP with water contact angle of 80°, and PMMA with water contact angle of ~65°), PHMB, B, G, and LS moistened the surfaces better compared to water, and the greatest reduction in water contact angle (30°) was observed for LS, for all the polymer-based substrates.

**Figure 4:**
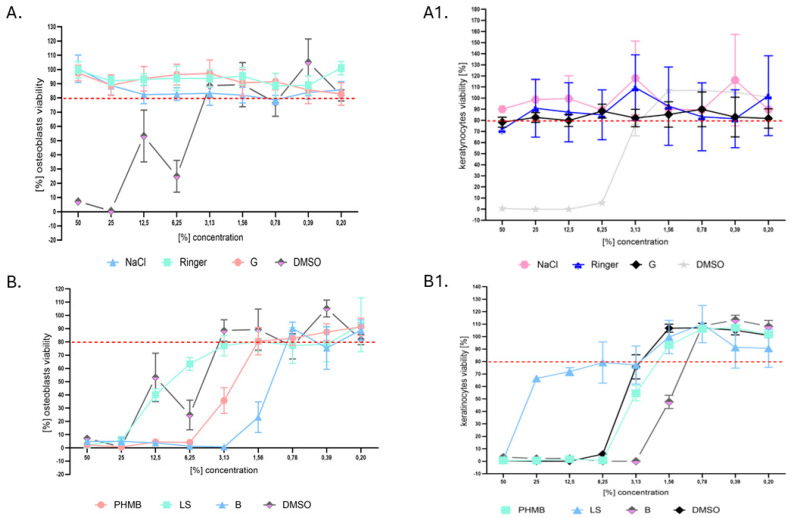
The [%] cytotoxicity of analyzed antiseptics/lavaseptics. (**A**,**A1**) Liquids displaying no cytotoxicity towards osteoblasts and keratinocytes, respectively: NaCl, Ringer’s solution, G, and cytotoxic concentration of DMSO used as a control setting. (**B**,**B1**) Liquids displaying dose-dependent cytotoxicity towards osteoblasts and keratinocytes, respectively: PHMB, LS, B, and DMSO used in a control setting. The red dashed line indicates the level (80% of viability) above which products are considered non-cytotoxic. NaCl: saline; PHMB: 0.1% polyhexanide + poloxamer surfactant; G: low-concentration hypochlorite; LS: 0.04% polyhexanide + macrogol surfactant; R: Ringer’s solution; B: iodine-containing antiseptic.

Because, in orthopedic surgery, the balance between achieving effective antimicrobial activity and avoiding potential harm to the patient’s tissues and cells is critical for treatment success, in the next analysis, the cytotoxicity of the investigated liquids towards osteoblasts and keratinocytes in vitro was assessed (Figure 4).

It can be observed that not only did the NaCl and R solutions, which do not contain antimicrobial components, not display significant cytotoxicity towards keratinocytes and osteoblasts (more than 80% of cells survived exposure), but neither did the G liquid, which contains low-concentration hypochlorite. The observed standard deviations of ca. 10% from average values were a result of the applied methodology, which includes a series of rinsings and might result in a random removal of cellular clusters. Regardless of the cell line tested, the same concentrations of PHMB displayed higher levels of cytotoxicity than LS; in turn, both these liquids displayed lower levels of cytotoxicity in the same concentration applied than the iodine-containing B antiseptic. The latter liquid stopped to be cytotoxic towards both osteoblasts and keratinocytes when it was diluted 128 times, while the PHMB antiseptic displayed non-cytotoxicity towards osteoblasts and keratinocytes when it was diluted 32 and 64 times, respectively. In turn, LS reached a non-cytotoxic level towards osteoblasts and keratinocytes when it was diluted 16 and 32 times, respectively (Figure 4).

**Figure 5:**
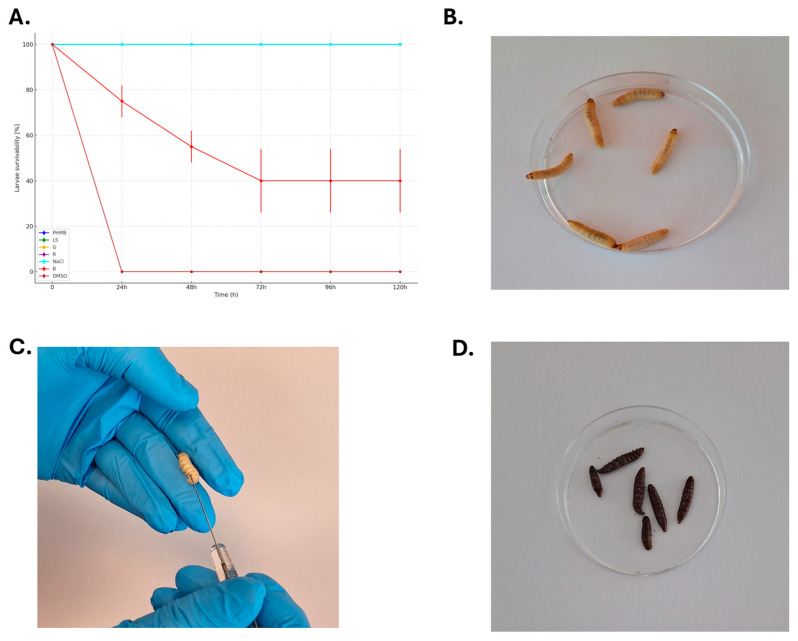
(**A**) The cytotoxicity [%] of larvae injected with the tested antiseptics and lavaseptics. As PHMB, LS, G, R, and NaCl displayed no cytotoxic effect on larvae during the experiment, their respective curves overlap and follow the same pattern observed for NaCl in the plot. The picture in (**B**) shows the process of the injection of the antiseptic into the interior of larvae. The picture in (**C**) shows live, motile *G. mellonella* larvae injected with saline after 5 days of incubation, while picture (**D**) shows dead, melanized, motionless *G. mellonella* larvae injected with DMSO 24 h post exposure. NaCl: saline; PHMB: 0.1% polyhexanide + poloxamer surfactant; G: low-concentration hypochlorite; LS: 0.04% polyhexanide + macrogol surfactant; R: Ringer’s solution; B: iodine-containing antiseptic; DMSO: dimethyl sulfoxide. The Kaplan curves for LS, G, R, NaCl overlap with PHMB curves (no cytotoxicity detected), therefore PHMB curve is representative for these 4 curves.

Normative cytotoxicity assays, as the one presented above, do not reflect the complexity of the interactions occurring within tissues. Therefore, in the final investigation line, the cytotoxicity of the tested antiseptics and lavaseptics was measured in a live model organism, namely larvae of *G. mellonella* (Figure 5).

The data obtained in the in vivo larvae model indicate that the injection of liquids at a weight equal to 10% of the larvae’s body mass contributed to significant morbidity only in the case of the iodine-containing B antiseptic. No larvae died within the 5-day period of observation after the application of the PHMB or LS liquids, contrary to the data shown in Figure 4.

## 3. Discussion

The rationale behind this publication stems from the urgent clinical need to provide new data on the potential use of antiseptics and lavaseptics in perioperative and postoperative care in orthopedic surgery. Infections caused by the patient’s own microbiota or microorganisms from contaminated instruments and/or introduced biomaterials can lead to the need for re-surgery and further complications, which not only significantly deteriorate the patient’s health but also place a considerable burden on healthcare systems [14,15].

In our study, we selected specific microbial species based on their clinical relevance to orthopedic infections. *S. aureus* is a leading cause of osteomyelitis and septic arthritis and is known for its ability to form biofilms on orthopedic implants, contributing to persistent infections [16]. *S. epidermidis*, a coagulase-negative staphylococcus, significantly contributes to prosthetic joint infections due to its biofilm-forming capabilities, leading to chronic and persistent infections [17]. *C. albicans*, a fungal pathogen, is associated with periprosthetic joint infections, particularly in immunocompromised patients, and has the ability to form biofilms on prosthetic materials, complicating treatment and eradication [18]. *C. acnes*, formerly known as *P. acnes*, is a notable cause of delayed-onset prosthetic joint infections, especially in shoulder arthroplasties, due to its indolent nature and biofilm-forming ability [19]. *P. aeruginosa*, known for its resistance to multiple antibiotics, is associated with post-surgical infections and can complicate open fractures, particularly in immunocompromised patients [20]. Although less commonly reported, *C. amycolatum* has been identified as a pathogen in orthopedic infections, including prosthetic joint infections, especially in immunocompromised individuals [21]. By including these clinically relevant microbial species, our study aimed to provide comprehensive insights into the effectiveness of antiseptic agents against pathogens commonly encountered in orthopedic infections.

In this study, we tested locally applied solutions containing (PHMB, LS, G, B) or not containing (R, NaCl) antimicrobial components. All these liquids are successfully used in the treatment of chronic wounds. Their beneficial effects rely either solely on mechanical rinsing (R, NaCl) or on a combination of mechanical rinsing and antimicrobial activity (PHMB, LS, and B). In the case of G, which contains low concentrations of hypochlorite, its antimicrobial efficacy remains controversial, as was previously shown [22]. Also, clinical data suggest that hypochlorite needs to be applied at concentrations several hundred times higher than those found in G if it is to be effective against microbial biofilms, as recently demonstrated by Fazli and Bjarnsholt [23].

Herein, we primarily focused on the activity of the aforementioned liquids against biofilms that, in a clinical context, develop on orthopedic biomaterials and surrounding tissues over an extended period of time. However, we recognize that, perioperatively, contaminating microorganisms might also form small, adhered clusters on these surfaces, rather than fully developed, multilayer biofilm structures [24]. Despite this, in vitro assessments targeting such small microbial populations may lead to false-positive results, suggesting that virtually every antiseptic or lavaseptic could effectively remove microorganisms and secure the surgical site. Therefore, we adopted the approach that if the tested liquids demonstrate activity against mature biofilms, they should exhibit significantly stronger effects against smaller microbial clusters.

Building on our previous experience [22,25,26,27], we employed a variety of experimental models in this work, incorporating both static and flow conditions, different surfaces for biofilm growth (including both biotic and abiotic ones), and varying exposure times. Additionally, aware of the major concern regarding the potential cytotoxicity of topical antimicrobial liquids towards eukaryotic cells, we also investigated this aspect using both in vitro and in vivo models.

The first line of investigation covered a standard microtitrate plate model for the assessment of the antimicrobial (understood here as “anti-planktonic” and expressed by MIC value) and antibiofilm (expressed in form of MBEC value) activity of the analyzed antiseptics/lavaseptics [Table 1]. This in vitro model is associated with significant disadvantages regarding the translation of the obtained results into clinical practice [28] due to the inadequate time of exposure to antimicrobials (24 h), the application of diluted antimicrobials, and flat polystyrene surfaces. Nevertheless, due to the widespread historical use of this approach, its use might be considered a necessary first step.

NaCl and R, which contain no antimicrobial components, had no effect on the microorganisms analyzed, similar to G. This lack of effect was observed for both planktonic cells and biofilms of the tested microorganisms. For all microorganisms and liquids tested, the recorded MBEC values exceeded the MIC values by several to a dozen times, clearly demonstrating the well-recognized increased tolerance of biofilms to antimicrobials [29]. As shown in Table 1, PHMB, and even LS (which contains 2.5 times lower concentrations of polyhexanide than PHMB), could be diluted more than the iodine-containing B and still effectively kill the tested pathogens. On the other hand, the differences between the MIC and MBEC values for the B liquid were smaller (more favorable), ranging from 2 to 8 times, compared to PHMB, where the difference between these values ranged from 4 to 16 times.

The next test, dubbed B.O.A.T. (Biofilm-Oriented Antiseptic Test), allows for the application of undiluted liquids in more relevant, clinically oriented contact times [Table 2]. As shown, along with the exposure of biofilms to the tested liquids, the percentage of eradicated biofilm-forming strains increased as well. Contrary to the results presented in Table 1, where the PHMB liquid displayed the highest activity, in the B.O.A.T. test, the B liquid was able to completely eradicate biofilms formed by all pathogens in the longest (24 h) time applied, and the biofilm of *P. aeruginosa* in 30’ of contact time, which did not occur when PHMB was applied. This difference may be explained by the fact that iodine’s efficacy is highly concentration-dependent, as observed in our study. At low concentrations, iodine’s activity is significantly reduced, which can be attributed to several factors. First, iodine displays a non-linear dose–response relationship, meaning that only at sufficiently high concentrations can enough free iodine molecules interact with microbial components to initiate rapid cell damage. This suggests that iodine needs to exceed a critical threshold to fully exert its antimicrobial effects [30]. Second, iodine’s “activity threshold” is essential for its effectiveness. At sub-threshold levels, there may not be enough reactive iodine species to disrupt microbial cell membranes, proteins, or nucleic acids, allowing the microorganisms to potentially repair any minor damage inflicted. Moreover, iodine is susceptible to inactivation in the presence of organic material, as it rapidly reacts with proteins, lipids, and other cellular components, reducing its bioavailability and antimicrobial potency over time. This explains why iodine’s antimicrobial effect can diminish rapidly after its initial application, also in clinical settings, where contact with organic matter is common [30]. Finally, in the case of biofilms, microbial cells are further protected by the extracellular matrix, which limits iodine’s penetration. While high concentrations of iodine can overcome these protective mechanisms, lower concentrations are often insufficient to breach the biofilm, allowing microorganisms to survive and persist.

The remaining liquids (G, LS, R, NaCl) did not display any measurable antimicrobial activity. However, it should be noted that in this study, due to the multitude of strains and tests applied, only the qualitative version of the B.O.A.T (indicating whether complete eradication did or did not occur) was conducted. A quantitative version of the B.O.A.T. could provide a more nuanced dataset, showing the percentage [%] of reduction compared to an untreated control group [26].

After completing the assessment of polystyrene-based models, the soft, tissue-like surface of bacterial cellulose (BC) was used to culture microorganisms [Figure 2]. The rationale was that BC would mimic the tissue surrounding bone or biomaterials. Its scaffold-like structure allows microorganisms to penetrate its interior, providing additional protection against antiseptics. Interestingly, in this model, PHMB showed significantly higher (*p* < 0.0001) efficacy than B, despite the opposite outcome observed in the previous B.O.A.T. test. This discrepancy could be due to PHMB’s ability to penetrate and interact more effectively with the scaffold structure of BC, reaching deeper-embedded microorganisms. In contrast, B, which relies on iodine, may be less effective due to the physical absorption in BC, which could trap iodine within the material, limiting its contact with microorganisms [31]. The ease of iodine absorption is the reason why antiseptics based on this element are avoided in wound treatment for patients with thyroid conditions or premature infants [32]. In turn, G, LS, R, and NaCl displayed significantly lower activity (*p* < 0.0001) compared to PHMB and B, with their efficacy being comparable regardless of whether the liquid contained antimicrobial components or not. This low level of eradication can once again be attributed to the rinsing procedures used in this technique rather than to the antimicrobial or lavage properties of these liquids.

The data in Figure 2 show the level of eradication achieved when biofilms grown on orthopedic-relevant biomaterials were exposed to the tested liquids for 5 min. These results are the outcome of at least two factors: the antimicrobial activity itself (understood as the ability to kill microorganisms) and the rinsing force generated by the applied surfactant, as all other conditions were kept constant. The outcomes obtained were fully consistent with those from the BC model, where PHMB exhibited significantly stronger activity than B, which in turn was significantly stronger than G, LS, R, and NaCl. LS displayed antimicrobial activity at a level similar to or slightly higher than G. Therefore, emphasis should also be placed on the third factor, namely the interplay between the antimicrobial agent and the surfactant. One may hypothesize that a fast-acting antimicrobial, such as PHMB or iodine [33], may disrupt the biofilm structure by destroying cells, which facilitates the biofilm’s detachment by the surfactant. On the other hand, the surfactant, by loosening the extracellular matrix structure, may enhance the accessibility of the biocidal component to the cells [34].

The minimal biofilm disruption with NaCl and Ringer’s solution depicted in Figure 2 likely results from the mechanical forces applied during rinsing rather than any inherent chemical effects on the biofilm. This underscores the significance of physical rinsing in biofilm management, especially in surgical contexts, where the mechanical removal of debris and microorganisms is crucial for infection control. Interestingly, low-concentration hypochlorite demonstrated a similar level of biofilm removal as NaCl and Ringer’s solution. This suggests that, at lower concentrations, hypochlorite functions more as a lavage agent rather than as an antiseptic, aligning with previous discussions on its role. This finding highlights the importance of considering both the concentration and intended function of hypochlorite in clinical applications.

The observed reduced efficacy of antiseptics in removing biofilms from orthopedic biomaterials (stainless steel screws, Ti-Al-Nb scaffolds), compared to the disks made from the same material, can be attributed to the more complex surface topographies of the former. These surface irregularities provide additional protection for biofilms by creating niches where microorganisms can attach, proliferate, and form more resilient biofilm structures [35]. In contrast, metal or alloy disks typically have smoother, less complex surfaces, allowing antiseptics to interact more directly with the biofilm. To provide translational insight, the increased surface area and the complexity of orthopedic biomaterials not only shield biofilms from direct contact with antiseptics but may also reduce the effectiveness of mechanical forces during rinsing, further limiting the removal of biofilms. This indicates that the structural properties of orthopedic biomaterials play a crucial role in biofilm persistence and highlight the need for the use of efficiently acting antiseptics in such applications.

At this moment, it is difficult for us to explain the results presented in Figure 3, where LS exhibited visibly stronger activity against *S. aureus* than PHMB, despite containing 2.5 times lower concentrations of polyhexanide. Some insight was provided by wettability measurements where LS demonstrated the most extensive spreading compared to the other tested liquids. The low contact angle recorded for LS may translate into a high surface wetting ability, potentially contributing to greater removal efficiency, especially in such narrow cylindrical interiors as those in the microcapillaries. In contrast, the bulk of red-dyed cells clogging the microcapillary after exposure to the iodine-containing B product is most likely due to iodine’s mechanism of action, which involves rapid cell denaturation and dehydration. This process may cause the organic remnants of the cells to adhere to the microcapillary. Overall, qualitative data from microfluidic model indicated a higher efficacy of LS than PHMB and B, while these latter two antiseptics acted more efficiently than G, R, and NaCl.

Polyhexanide-based solutions (PHMB and LS) exert their antimicrobial effect by disrupting bacterial cell membranes through electrostatic interactions, leading to the leakage of cellular contents. They also demonstrate antibiofilm activity on both simple and complex surfaces. In turn, povidone–iodine is a broad-spectrum antimicrobial agent that oxidizes microbial proteins, nucleotides, and membrane lipids, resulting in rapid cell death. Its efficacy is highly dependent, with limitations in biofilm penetration and potential cytotoxic effects. Hypochlorite (G) acts by generating reactive oxygen species that cause oxidative damage to microbial DNA, proteins, and lipids. However, its antibiofilm activity at low concentrations is minimal, though it also displays low cytotoxicity. Saline and Ringer’s solutions (NaCl and R) serve primarily as mechanical rinsing agents without intrinsic antimicrobial properties. Their main function is to flush out debris and biofilm through mechanical disruption rather than chemical action.

In the investigation of eukaryotic cells presented in this work, a standard cytotoxicity test was conducted [Figure 4]. This experimental setup shares several limitations with the microtiter plate assay on microorganisms described in Figure 1, such as irrelevant exposure times and the use of flat, abiotic surfaces. Despite these drawbacks, this in vitro cytotoxicity test remains commonly used; however, it is increasingly being replaced by more advanced assessments, such as those based on matrigel, which offer a more biologically relevant environment. Herein, we applied osteoblast cell lines due to their orthopedic relevance, and keratinocytes were also considered for cytotoxicity studies. Given that antimicrobial compounds may encounter both bone and surrounding soft tissues during surgery, assessing their effects on keratinocytes could offer a broader safety profile. This complements previous studies by Kramer and Severing [36,37], who investigated fibroblasts in this context, making keratinocytes a logical next step for evaluating potential impacts on skin tissue. The results suggest that antiseptics such as PHMB and the iodine-containing antiseptic B, while effective against biofilms, display significant cytotoxicity toward osteoblasts and keratinocytes at higher concentrations in vitro. In contrast, exposure to G, NaCl, and R did not reduce cell viability below 80%, which is considered a safe threshold. Notably, the concentrations of PHMB that were effective against planktonic bacteria were approximately 2–4 times higher than those that were non-cytotoxic to eukaryotic cells, with an even greater discrepancy observed for biofilm eradication. The iodine-containing antiseptic B exhibited even higher cytotoxicity than PHMB at antimicrobial concentrations [compare Table 1 vs. Figure 4]. These findings underscore the importance of rinsing such antiseptics after clinical application to prevent cellular damage, although their strong biofilm removal capacity could mitigate the risk of severe infectious complications.

A cytotoxicity test conducted in vitro does not provide the cells with protection from the extracellular matrix or other factors present in living tissues. Therefore, in the final stage of our research, we injected antiseptics/lavaseptics into *G. mellonella* larvae [Figure 5]. In the context of assessing the toxicity and effectiveness of antiseptics, this model is useful because the larvae’s immune system exhibits certain similarities to the mammalian one, although it is much simpler [38]. Additionally, the larvae allow for quick, cost-effective in vivo studies, and their viability indicators, such as color changes or locomotor activity, can be easily monitored, providing valuable data on the potential effects of antiseptics [39]. The results from the in vivo larvae model revealed that injecting liquids amounting to 10% of the larvae’s body mass caused significant morbidity only in the case of the iodine-containing antiseptic B, while no deaths were observed over a 5-day period following the application of the PHMB or LS liquids. This contrasts with the in vitro cytotoxicity data, where PHMB also appeared harmful to cells [Figure 4]. The discrepancy can be attributed to the protective role of the extracellular matrix (ECM) and tissue-specific factors in living organisms, which are absent in in vitro conditions. The ECM helps shield cells from harmful substances and modulates their interactions with antiseptics [40]. In vivo, PHMB’s toxicity may be mitigated by these natural defenses, resulting in less cellular damage [41]. However, the iodine-containing antiseptic B remains highly cytotoxic due to iodine’s strong oxidative properties, which can disrupt cellular components like proteins and lipids, overwhelming the ECM’s protective capacity. This explains why the iodine-based antiseptic B showed significant toxicity in the larvae model, underscoring the importance of cautious clinical use, particularly the need to rinse iodine-based antiseptics to prevent damage to bone tissues, despite their strong antimicrobial effects [42].

This investigation explored a diverse range of experimental models to evaluate the effects of locally applied antiseptics and lavaseptics on biofilms and eukaryotic cells. One key finding presented in this manuscript is that low-concentration hypochlorite lacks significant antimicrobial activity (i.e., the ability to kill microorganisms) and should be classified as a lavaseptic by definition. Another important observation is that a solution containing 0.04% polyhexanide is less effective against biofilms (both antiseptically and lavaseptically) compared to a 0.1% polyhexanide solution (observed in 4 out of 5 experimental models). However, the 0.04% solution also exhibited lower cytotoxicity in vitro and similar cytotoxicity in vivo to its more concentrated counterpart. Notably, the 0.1% polyhexanide solution outperformed the iodine-containing antiseptic when tested on more complex biofilm surfaces, whereas the iodine-containing antiseptic was more effective in standard microdilution assays. Another translational outcome from our experiments is the observation that although in vivo tests showed that antiseptics may not be as cytotoxic as suggested by standard polystyrene-based assays, it is still recommended to rinse antiseptics off after their application on bone tissue or orthopedic materials [43]. An open question remains regarding the optimal duration of exposure of bone and soft tissue to antiseptics and the appropriate amount of liquid required for effective rinsing.

Data from our study suggests that NaCl and Ringer’s solution can be safely used for this purpose, though their application is insufficient to fully remove microorganisms from colonized surfaces.

In conclusion, this study highlights the complexity and significance of evaluating antiseptics and lavaseptics for clinical use, particularly in orthopedic surgery, where the prevention and treatment of biofilm-related infections are critical. Our findings underscore the need for a diversified experimental approach, incorporating both in vitro and in vivo models, to capture the multifaceted interactions between antimicrobial agents, biofilms, and host tissues. While in vitro tests remain essential for initial screening, they often fail to account for protective mechanisms, such as the extracellular matrix, which can mitigate cytotoxic effects. The discrepancies between our in vitro cytotoxicity results and those from the in vivo *G. mellonella* model further demonstrate the importance of this comprehensive approach. Key discoveries from this investigation include the identification of high concentrations of iodine-containing antiseptic B as cytotoxic, despite its strong antimicrobial action. Conversely, PHMB demonstrated lower toxicity and comparable biofilm-eradication efficacy, especially on complex surfaces. These insights suggest the need for careful consideration of antiseptic application protocols in clinical settings, including thorough rinsing to avoid tissue damage while maximizing biofilm removal.

One of the primary limitations of this study is the inherent discrepancy between in vitro and in vivo models, which may not fully replicate the complex interactions that occur in a clinical setting, particularly within human tissues. While in vitro experiments provide valuable initial insights into the antimicrobial and cytotoxic properties of antiseptics, they do not account for factors such as the extracellular matrix, immune responses, or blood flow, which can significantly influence the behavior of these agents in vivo. However, we attempted to mitigate this limitation by incorporating an in vivo model using *G. mellonella* larvae, which, while simpler than mammalian models, offers valuable translational data due to its similarities with the mammalian immune system.

Future research should focus on refining the use of these antiseptic agents, optimizing exposure times, and developing improved rinsing strategies. Moreover, the continued exploration of alternative models and surfaces will be crucial to better understand the real-world implications of antiseptic and lavaseptic use in surgical environments. This will guide the development of more effective infection control measures that balance antimicrobial potency with patient safety.

## 4. Materials and Methods

### 4.1. Antiseptic and Lavaseptic Products Analyzed

(a) Polyhexanide-containing product under brand name Preventia Surgical Irrigation Solution (Paul Hartmann AG, Heidenheim an der Brenz, Germany) containing, according to the manufacturer, polyhexanide (1.0 g), and poloxamer, later referred to as “PHMB”.
(b) Hypochlorous acid/sodium hypochlorite-based product under brand name Granudacyn Wound Irrigation Solution (Molnycke, Goteburg, Sweden) containing, according to the manufacturer, water, sodium chloride, hypochlorous acid (0.05 g), and sodium hypochlorite (0.05 g), later referred to as “G”.
(c) Polyhexanide-based product under brand name LavaSorb (B. Braun Medical AG, Melsungen, Germany) containing, according to the manufacturer, polyhexanide (0.40 g), macrogol 4000 (0.02 g), sodium chloride (8.60 g), calcium chloride dihydrate (0.33 g), potassium chloride (0.30 g), and purified water up to 1 L, later referred to as “LS”.
(d) Povidone–iodine-containing product under brand name Betasoidona (Hermes Artzneimittel, Pullach im Isartal, Germany), containing, according to the manufacturer, povidone–iodine (100 mg), equivalent to iodine (11 mg), and excipients including glycerol, nonoxynol-9, disodium phosphate, citric acid, sodium hydroxide for pH adjustment, and potassium iodate in 1 mL of solution, later referred to as “B”.
(e) Saline, 0.9% (B. Braun Medical AG, Melsungen, Germany), later referred to as “NaCl”.
(f) Ringer’s solution (B. Braun Medical AG, Melsungen, Germany), containing, according to the manufacturer, sodium chloride (8.6 g), potassium chloride (0.3 g), and calcium chloride dihydrate (0.33 g/L) in 1 L of water for injections, later referred to as “R”.

### 4.2. The Organisms

This study was carried out on bacterial and fungal strains from the Collection of The Department of Pharmaceutical Microbiology and Parasitology, Medical University of Wroclaw, Poland, and reference strains from the American Tissue and Cell Culture Collection ATCC (Manassas, VA, USA). The investigated group of microorganisms consisted of the following:

(a) *Staphylococcus epidermidis*, (n = 25), including ATCC strain number 12228;
(b) *Staphylococcus aureus* MRSA (n = 25), including ATCC strain number 33591;
(c) *Cutibacterium acnes* (n = 25), including ATCC strain number 11828;
(d) *Corynebacterium amycolatum* (n = 25), including ATCC strain number 700207
(e) *Pseudomonas aeruginosa* (n = 25), including ATCC strain number 27853;
(f) *Candida albicans* (n = 25), including ATCC strain number 10231;
  Clinical strains isolated from chronic leg ulcers and long-bone infections under approval of the Bioethics Committee of Wroclaw Medical University, number 949/2022;
(g) Cellulose-forming *Komagataeibacter xylinus* number 53524;
(h) *Galleria mellonella* larvae, bred and cultivated in “P.U.M.A.” Platform for Unique Model Application, applied for the in vivo tests. The larvae applied in experiments were of 200 ± 10 mg weight.

### 4.3. Surfaces Applied for Biofilm Development and Subsequent Removal Using the Provided Antiseptic/Lavaseptic Products

(a) Disks with 16 mm diameter/2 mm height made of medical-grade polystyrene (Compamed, Dusseldorf, Germany), later referred to as “PS”. This surface, not applied in orthopedic settings but commonly used as a reference surface for biofilm growth in vitro, was applied in the character of control setting.
(b) Stainless-steel disks with 16 mm diameter/2 mm height (Kamb, Warsaw, Poland), later referred to as SSDs.
(c) Co-Cr-Mo disks with 16 mm diameter/2 mm height (Schutz Dental, Rosbach vor der Hoche, Germany), later referred to as Co-Cr-Mo.
(d) Ti-Al-Nb disks with 16 mm diameter/2 mm height (Kamb, Warsaw, Poland), later referred to as Ti-Al-Nb-D.
(e) Ti-Al-Nb scaffold implants with 8 mm diameter/8 mm height (Kamb, Warsaw, Poland) produced by Additive Manufacturing SLM processing, as described in an earlier work of ours [44], later referred to as Ti-Al-Nb-S.
(f) Ultra-high-molecular-weight polyethylene disks with 16 mm diameter/2 mm height (Enimat, Bydgoszcz, Poland), later referred to as UHMPWEs.
(g) Orthopedic stainless-steel screws with a size of 6 mm × 1.8 mm (Biomedent, Houston, TX, USA), later referred to as SSCs.
(h) Bacterial cellulose disks with 16 mm diameter/2 mm height, later referred to as BC, obtained as described earlier [45]. *K. xylinus* strain ATCC 53524 was incubated in Hestrin–Schramm medium (2% glucose (*w*/*v*; Chempur, Piekary Slaskie, Poland), 0.5% yeast extract (*w*/*v*; VWR Chemicals, Radnor, PA, USA), 0.5% bactopeptone (*w*/*v*; VWR Chemicals, Radnor, PA, USA), 0.115% citric acid (*w*/*v*; POCH, Gliwice, Poland), 0.27% Na_2_HPO_4_ (*w*/*v*; POCH, Gliwice, Poland), 0.05% MgSO_4_ × 7H_2_O (*w*/*v*; POCH, Gliwice, Poland), and 1% ethanol (*v*/*v*; Stanlab, Lublin, Poland)) for 7 days at 28 °C in 24-well microtiter plates (F type, Nest Scientific Biotechnology, Wuxi, China). Next, the formed BC disks were removed from the medium and cleansed using 0.1 M NaOH (Chempur, Piekary Slaskie, Poland) solution at 80 °C until the BC became white. Then, the BC disks were purified using water until they reached a pH of 7 (measured by pH strips, Macherey–Nagel, Düren, Germany) and sterilized in a steam autoclave. The BC disks were placed into 24-well plates (F type, Nest Scientific Biotechnology, Wuxi, China) in sterile water and incubated at 4 °C until the time of further experiments.

### 4.4. Measurement of Minimum Biocidal Concentration (MBC) and Minimum Biofilm Eradication Concentration (MBEC) of Antiseptics and Lavaseptics Using Microtitrate Plate Model

(a) MBC: Microbial suspensions at a density of 0.5 McF (McFarland turbidity scale) (Densitomat II, BioMerieux, Warsaw, Poland) in 0.9% NaCl (Stanlab, Lublin, Poland) were prepared from fresh, 24 h cultures in appropriate culture broths (M-H for *S. aureus*, *S. epidermidis*, *P. aeruginosa*, *C. albicans*; BHI for *C. acnes*). All media were purchased from Graso, Jablowo, Poland. The suspensions were diluted 1000 times in appropriate media to reach ~10^5^ cfu/mL and 100 μL of each suspension was introduced into 10 wells of a 96-well plate (VWR, Radnor, PA, USA). Next, serial dilutions of antiseptics/lavaseptics were added to each of well containing the microbial suspension. Therefore, the highest *v*/*v* concentration of the antimicrobial was 50%, while the lowest was 0.1%. The control setting of bacterial/fungal growth (microbial suspension without antiseptic/lavaseptic) and sterility control (broth without suspended microbes) were provided in wells number 12 and 11, respectively. The plates containing *S. epidermidis*, *S. aureus*, *C. amycolatum*, *P. aeruginosa*, and *C. albicans* were incubated for 24 h at 37 °C under stationary conditions in aerobic conditions, while *C. acnes* was incubated for 24 h/37 °C in anaerobic conditions provided by an anaerobic atmosphere generation container (Sigma-Aldritch, Taufkirchen, Germany). Analyses on *C. acnes* were performed with the use of an anaerobic chamber (Jacomex, Dagneux, France). The next day, a standard resazurin sodium salt solution (Acros Organics, Geel, Belgium) was applied to indicate the concentration of product, which caused the stop of metabolic activity. Wells from parallel prepared plates with no resazurin solution were introduced into 9800 µL of the appropriate medium and incubated for 72 h. If no turbidity change occurred, the specific concentration of the antiseptic/lavaseptic used was considered MBC.
(b) MBEC: Microbial suspensions at a density of 0.5 McF (McFarland turbidity scale) (Densitomat II, BioMerieux, Warsaw, Poland) in 0.9% NaCl (Stanlab, Lublin, Poland) were prepared from fresh, 24 h cultures in appropriate culture broths (M-H for *S. aureus*, *S. epidermidis*, *P. aeruginosa*, *C. albicans*; BHI for *C. acnes*). All media were purchased from Graso, Jablowo, Poland. The suspensions were diluted 1000 times in appropriate media to reach ~10^5^ cfu/mL and 100 μL of each suspension was introduced into 10 wells of a 96-well plate (VWR, Radnor, PA, USA). Next, the plates were incubated for 24 h (*S. aureus*, *S. epidermidis*, *P. aeruginosa*, *C. albicans*) or 48 h (*C. acnes*) in the conditions specified in the MBC assessment. After incubation and medium removal, serial dilutions of the antiseptics/lavaseptics were added and plates were subjected to another 24 h of incubation. Next, the medium was removed, neutralizing agents were applied for 5 min, and after that, the procedures related to resazurin staining and transfer to fresh media were performed as described in the protocol for MBC assessment.

### 4.5. Measurement of Activity of Antiseptics/Lavaseptics Against Biofilms Using B.O.A.T. (Biofilm-Oriented Antiseptic Test)

This method was performed according to our earlier published work [26]. The biofilm was established as described in Section 4.4 of Materials and Methods (MBEC). Next, the media were removed and 100 μL of antiseptic/lavaseptic was added to the well with adhered biofilms. The contact times were 30’, 1 h, and 24 h, while the incubation temperature was 37 °C. After exposure, the antiseptics/lavaseptics were gently removed and 100 μL of the appropriate neutralizer was introduced for 5 min at room temperature. Then, the neutralizers were removed and 100 μL of the appropriate medium was added. The plates with pre-established biofilms of *S. aureus*, *S. epidermidis*, *P. aeruginosa*, and *C. albicans* were incubated for 24 h/37 °C, while the biofilm of *C. acne* for 48 h. The survival/eradication of biofilms was confirmed by a change in turbidity and by the measurement of metabolic activity using tetrazolium salt assay or the spot method. Ten strains of each species were selected on the basis of their median MBEC values assessed in the standard microtitrate model described in Materials and Methods Section 4.4. The BOAT test was performed in 6 repeats.

### 4.6. The Activity of Antiseptics/Lavaseptics Tested in Bacterial Cellulose Model

The antiseptics/lavaseptics were used on the biofilms formed on bacterial cellulose carriers according to our earlier published work [22]. BC carriers of 18 mm diameter were soaked in MH (BHI broth; Biomaxima, Lublin, Poland) medium overnight at 8 °C. The next day, the BC carriers were transferred to 24-well plates (VWR, Radnor, PA, USA). A 0.5 McF suspension of microorganisms, diluted 1000 times, was prepared in BHI medium, and 1 mL of this suspension was added to each BC carrier and subjected to stationary incubation for 48 h at 37 °C. Next, the suspension was removed, and the BC carriers were transferred to fresh 24-well plates. A total of 1 mL of undiluted antiseptic/lavaseptic agent was added to the BC containing a microbial biofilm and left for 30 min at room temperature. Next, the antiseptic/lavaseptic products were removed and 1 mL of neutralizer was added to each well for 5 min. Next, the neutralizers were removed and 1 mL of 0.1% saponine was added. The whole setting was subjected to intense vortex shaking for 1 min. After that, the obtained suspension was subjected to quantitative culturing on appropriate agar plates. This analysis was performed in 6 repeats. The number of cells from the biofilm exposed to the presence of water instead of antiseptic/lavaseptic products was considered 100% of potential cellular growth in this setting.

### 4.7. The Activity of Antiseptics/Lavaseptics Against Biofilms Preformed on Biomaterials in Flow Conditions

The ability of the antiseptic/lavaseptic products to flush biofilms out of the orthopedic surfaces listed in Section 4.3 point a–g was determined as shown in an earlier work of ours [46]. Flow conditions provided by the Ismatec Reglo digital peristaltic pump (Randor, PE, USA) were used to enable the microbial adhesion of 10^5^ cells/mL on the orthopedic surfaces for 1 h; next, the biofilms developed for 48 h in static conditions. Next, the antiseptic/lavaseptic agents were introduced into boxes containing the orthopedic surfaces for the applied contact time of 5’ at a pace of 1 mL/min. After that, the neutralizer was introduced in stationary conditions for 5’. The surfaces were exposed to 5 mL of 0.1% saponine and subjected to quantitative culturing on appropriate agar media. The number of cells from biofilms exposed to the presence of water and in no-flow conditions, instead of antiseptic/lavaseptic products, was considered 100% of potential cellular growth in this setting. This analysis was performed in 6 repeats.

### 4.8. Ability of Antiseptic/Lavaseptic Products to Flush Biofilm out Measured by Means of Microfluidic System

This analysis was performed towards *C. albicans*, *P. aeruginosa*, and *S. aureus* biofilms using a microfluidic BioFlux^®^ (Fluxion, San Francisco, CA, USA) model in an analogous way to the protocol presented in an earlier work of ours [25]. The microfluidic channels were flushed from inlet to outlet with TSB medium with a speed of 10 dyne/cm^2^ for 10 s. Next, 0.1 mL of microbial solution in TSB medium (OD600 = 1.0) was put into each of the outlet wells. The flow of microbe-containing solutions was turned on from the outlet towards the inlet wells at 5 dyne/cm^2^ for 5 s. The solutions were left for 1 h of incubation in 37 °C to enable adhesion to the microcapillaries’ surface. Subsequently, 0.9 mL of TSB medium was introduced to the inlet wells, and the medium flow was turned on from the inlet to outlet wells with an intensity of 0.5 dyne/cm^2^/24 h/37 °C. After culturing, both the inlet and outlet wells were drained. A total of 0.5 mL of the solution containing one of the tested products in a 1:1 ratio with TSB medium was then added to the inlet wells and directed for medium flow with a rate of 1.5 dyne/cm^2^. TSB medium was used in the control setting. Subsequently, the inlet wells were again emptied and filled with 0.1 mL of a saline solution with 0.3 μL of SYTO9 and 0.3 μL propidium iodide (Filmtracer™ LIVE/DEAD™ Biofilm Viability Kit; ThermoFisher Scientific, Waltham, MA, USA) for *S. aureus*, *P. aeruginosa*, and *C. albicans*. These solutions were passed through microcapillaries for 1 h in the outlet direction. Photographs of the microbial biofilms were taken with an inverted Carl Zeiss microscope (Carl Zeiss, GmbH, Jena, Germany). The degree of biofilm development, interpreted based on the microcapillaries’ coverage and the ratios of green/red fluorescence, which constituted information about the viability of the biofilms, was calculated using ImageJ software version 1.54k (NIH, Bethesda, WA, USA).

### 4.9. Wettability Measurement

In the study, several synthetic materials with different wettability were used, including the following: microscope slide (glass), PLLA plate (Resomer L210s, Evonik, Essen, Germany) processed by injection molding (Boy XS, Neustadt, Germany, injection pressure 180 bar, 200 °C), PLLA/hydroxyapatite (30 wt.% of the hydroxyapatite in the system) composite (Boy XS, injection pressure 200 bar, 200 °C), and PMMA plate (Altuglas V920, Resinex, High Vycombe, Great Britain). Water contact angle (WCA) measurements were carried out using a PGX pocket goniometer (Fibro system, AB, Stockholm, Sweden). For each material, at least 10 measurements were carried out at room temperature. As a reference, deionized water (water conductivity ~5–7 µS) was used.

### 4.10. Normative In Vitro Neutral Red (NR) Cytotoxicity Assay Towards Keratinocytes and Osteoblasts

The cytotoxicity test was performed towards ATCC 92022711 U2-OS osteoblasts and 300493 HaCaT keratinocytes (DKFZ, Heidelberg, Germany) in a 96-well micro-well plate model according to the ISO 10993-5:2009; Biological evaluation of medical devices—Part 5: Tests for in vitro cytotoxicity [47] as we described earlier [48]. The antiseptic/lavaseptic agents were introduced into the confluent cell cultures in a series of dilutions in such a manner that the highest concentration of the antiseptic/lavaseptic was 50% (*v*/*v*) of the working solution (undiluted product) and subjected to incubation for 24 h in 5% CO_2_ at 37 °C. As a control substance with known cytotoxic properties, 50% dimethylosulfate oxide (DMSO, ChemPur, Piekary Slaskie, Poland) was used. Next, the medium was removed and 100 μL of Neutral Red (NR) solution (40 μg/mL; Sigma-Aldrich) was introduced to the wells of the plate containing the cell lines. Subsequent incubation with NR lasted for 2 h at 37 °C. After this time, the NR was removed and the wells were rinsed with PBS (Phosphate-Buffered Saline, Biowest, Riverside, MO, USA) and left to dry at room temperature. A total of 150 μL of de-stain solution containing 50% ethanol, 49% deionized water, and 1% glacial acetic acid (*v*/*v*) (POCH) was then introduced to each well of the plate and vigorously shaken using a microtiter plate shaker (Plate Shaker-Thermostat PST-60HL-4, Biosan, Riga, Latvia) for 30 min until NR extraction. Next, the NR absorbance value was measured using a microplate spectrometric reader (Multi-scan GO, ThermoFisher Scientific) at a wavelength of 490 nm. The absorbance value of the cells not treated with the extracts was considered 100% of possible cellular growth (constituting positive control sample). The negative control was osteoblasts exposed to 50% ethanol (POCH) for 3 min. These analyses were performed in 6 repeats.

### 4.11. Analysis of Antiseptic/Lavaseptic Cytotoxicity In Vivo Towards G. mellonella Larvae Model

A larvae model was used to assess antiseptic/lavaseptic cytotoxicity in vivo. Larvae of the greater wax moth, *G. mellonella*, of average weight equal to 0.20 ± 0.2 g, were selected for the experiment. The larvae were injected with 20 μL of undiluted antiseptic/lavaseptic products (constituting 10% of larvae mass) to evaluate their cytotoxicity. The exception was the B antiseptic, where the introduced volume was decreased to 10 μL, because 20 μL led to the death of the larvae within the first 24 h of the experiment. Moreover, negative control with 20 μL of PBS (Biowest, Riverside, MO, USA) was used. The usability control was the injection of 10 μL of 96% (*v*/*v*) ethanol (Stanlab, Lublin, Poland). The larvae were placed in 90 mm Petri dishes (Noex, Warsaw, Poland) and incubated at 30 °C/five days. Each day, the mortality of the larvae was monitored. Death was defined when the larvae were nonmobile, melanized, and did not react to physical stimuli. Every antiseptic/lavaseptic product was tested in 10 larvae in two repeats, giving 20 larvae/product.

### 4.12. Statistical Analysis

Statistical analyses were performed using GraphPad Prism 10 (San Diego, CA, USA). The normality of distribution was verified using Shapiro–Wilk’s test. An Analysis of Variance (ANOVA) was performed to assess statistical significance. For multiple comparisons, Tukey’s post hoc test was applied. A *p*-value threshold of less than 0.05 was set for significance in the ANOVA. For the Tukey post hoc analysis, significance levels were further categorized as *p* < 0.001 and *p* < 0.0001 for specific pairwise comparisons.

## Figures and Tables

**Table 1:** Minimum Biocidal Concentrations [%] and Minimum Biofilm Eradication Concentrations [%] of tested products. Median values measured for 25 strains/species are presented. “none”—non-measurable within tested concentration range (0.1–50% of working solution) of antiseptics/lavaseptics. For higher transparency, table cells containing MBC/MBEC values are highlighted in blue, while table cells where no MBC/MBEC values were recorded are highlighted in green. NaCl: saline; PHMB: 0.1% polyhexanide + poloxamer surfactant; G: low-concentration hypochlorite; LS: 0.04% polyhexanide + macrogol surfactant; R: Ringer’s solution; B: iodine-containing antiseptic.

		PHMB	G	LS	B	NaCl	R
*S. epidermidis*	MBC	0.2%	none	1.56%	6.25%	none	none
	MBEC	3.13%	none	25%	50%	none	none
MRSA	MBC	0.1%	none	1.56%	6.25%	none	none
	MBEC	3.13%	none	25%	12.5%	none	none
*P. aeruginosa*	MBC	3.13%	none	6.25%	12.5%	none	none
	MBEC	12.5%	none	50%	50%	none	none
*C. albicans*	MBC	1.56%	none	6.25%	12.5%	none	none
	MBEC	6.25%	none	12.5%	50%	none	none
*C. acnes*	MBC	0.4%	none	0.8%	6.25%	none	none
	MBEC	6.25%	none	25%	50%	none	none
*C. amycolatum*	MBC	0.8%	none	3.13%	12.5%	none	none
	MBEC	6.25%	none	50%	50%	none	none

**Table 2:** B.O.A.T results. Strain survival of treatment for 30’, 1 h, and 24 h with the tested liquids is marked with “+”, while eradication as a result of the treatment is marked with “-”. The percentage values next to “+” refer to the situation when certain strains survived the treatment (while some of them did not) and indicate the percentage number of the strains survived out of 100% tested. For higher transparency, the exposure times in which a full eradication (100%) of biofilm-forming cells was observed are highlighted in blue; partial eradication is highlighted in orange; exposure times in which any of the tested biofilm-forming strains were eradicated fully are highlighted in green. NaCl: saline; PHMB: 0.1% polyhexanide + poloxamer surfactant; G: low-concentration hypochlorite; LS: 0.04% concentrated polyhexanide + macrogol surfactant; R: Ringer’s solution; B: iodine-containing antiseptic.

	PHMB			G			LS			NaCl			R			B		
Species/Time	30’	1 h	24 h	30’	1 h	24 h	30’	1 h	24 h	30’	1 h	24 h	30’	1 h	24 h	30’	1 h	24 h
***S. epidermidis* [n = 10]**	+	+[60%]	-	+	+	+	+	+	+	+	+	+	+	+	+	+	+[10%]	-
***S. aureus* [n = 10]**	+	+[50%]	-	+	+	+	+	+	+	+	+	+	+	+	+	+	+[20%]	-
***C. acnes* [n = 10]**	+	+[20%]	-	+	+	+	+	+	+	+	+	+	+	+	+	+	+[10%]	-
***C. amycolatum* [n = 10]**	+	+[70%]	+[10%]	+	+	+	+	+	+	+	+	+	+	+	+	+[40%]	+[20%]	-
***P. aeruginosa* [n = 10]**	+	-	-	+	+	+	+	+	+	+	+	+	+	+	+	+	-	-
***C. albicans* [n = 10]**	+	-	-	+	+	+	+	+	+	+	+	+	+	+	+	+	+[30%]	-

## Data Availability

All data necessary to replicate this study is provided within the manuscript. The raw dataset is available upon reasonable request from the corresponding authors.

## Supplementary Materials

The supporting information can be downloaded at https://www.mdpi.com/article/10.3390/ijms252312720/s1.
