# Supplementary material for: In Vitro and In Vivo Translational Insights into the Intraoperative Use of Antiseptics and Lavage Solutions Against Microorganisms Causing Orthopedic Infections
Source: Int J Mol Sci. 2024 Nov 26;25(23):12720. doi: 10.3390/ijms252312720 (PMC11641374; doi:10.3390/ijms252312720)

International Journal of
Molecular Sciences

Supplementary Material S1. Particular MBC and MBEC values of tested antiseptics and lavaseptics towards particular strains.

S. epidermidis / MBC

Strain	PHMB	LS	B
ATCC	0.1%	0.78%	6.25%
1	0.1%	0.39%	6.25%
2	0.05%	0.39%	3,12%
3	0.8%,	0.39%	6.25%
4	0.05%,	0.78%	6.25%
5	0.4%,	1.56%	6.25%
6	0.05%,	3.12%	6.25%
7	0.4%,	3.12%	3.12%
8	0.1%,	3.12%	6.25%
9	0.8%,	3.12%	3,12%
10	0.05%,	1.56%	6.25%
11	0.4%,	3.12%	6.25%
12	0.05%,	1.56%	6.25%
13	0.4%,	1.56%	6.25%
14	0.2%,	0.39%	6.25%
15	0.05%,	0.39%	6.25%
16	0.4%,	0.39%	6.25%
17	0.05%,	0.78%	6.25%
18	0.4%	1.56%	6.25%
19	0.05%,	0.39%	6.25%
20	0.4%,	0.39%	6.25%
21	0.05%	1.56%	6.25%
22	0.4%	1.56%	6.25%
23	0.8%	1.56%	6.25%
24	0.05%,	0.78%	6.25%

S.epidermidis/MBEC

Strain	PHMB	LS	B
**ATCC**	1.56%	6,25%	25%
**1**	1.56%,	6,25%	25%
**2**	1.56%,	6,25%	50%
**3**	1.56%	12,5%	50%
**4**	3.13%,	12,5%	25%
**5**	0.78%,	6,25%	25%
**6**	1.56%,	6,25%	50%
**7**	0.39%,	25%	50%
**8**	3.13%,	25%	50%
**9**	3.13%,	25%	50%
**10**	0.39%,	6,25%	50%
**11**	0.78%,	6,25%	50%
**12**	3.13%,	6,25%	25%
**13**	3.13%,	25%	50%
**14**	3.13%,	25%	50%
**15**	3.13%,	50%	50%
**16**	1.56%,	25%	50%
**17**	3.13%,	25%	50%
**18**	3.13%,	25%	50%
**19**	3.13%,	25%	50%
**20**	3.13%,	50%	25%
**21**	3.13%,	25%	50%
**22**	1.56%,	25%	50%
**23**	1.56%,	50%	50%
**24**	0.39%,	6,25%	50%

S. aureus / MBC

Strain	PHMB	LS	B
**ATCC**	0,1%	3,13%	6.25%
**1**	0,1%	1,56%	6,25%
**2**	0,1%	1,56%	6,25%
**3**	0,05%	1,56%	3,125%
**4**	0,1%	0,78%	3,125%
**5**	0,05%	0,78%	6,25%
**6**	0,05%	0,78%	6,25%
**7**	0,05%	0,78%	6,25%
**8**	0,1%	3,13%	6,25%
**9**	0,2%	1,56%	6,25%
**10**	0,1%	0,78%	6,25%
**11**	0,2%	0,78%	6,25%
**12**	0,05%	0,39%	6,25%
**13**	0,2%	0,39%	6,25%
**14**	0,1%	1,56%	6,25%
**15**	0,1%	0,78%	6,25%
**16**	0,1%	0,39%	6,25%
**17**	0,1%	1,56%	6,25%
**18**	0,1%	1,56%	6,25%
**19**	0,1%	0,39%	6,25%
**20**	0,1%	1,56%	6,25%
**21**	0,1%	1,56%	3,125%
**22**	0,1%	1,56%	6,25%
**23**	0,1%	0,39%	6,25%
**24**	0,1%	1,56%	6,25%

S. aureus / MBEC

Strain	PHMB	LS	B
**ATCC**	1,56%	6,25%	12,5%
**1**	0,78%	6,25%	12,5%
**2**	0,78%	12,5%	25%
**3**	1,56%	12,5%	25%
**4**	1,56%	3,25%	6,25%
**5**	1,56%	3,25%	6,25%
**6**	0,39%	25%	25%
**7**	1,56%	12,5%	25%
**8**	0,39%	25%	12,5%
**9**	1,56%	25%	25%
**10**	1,56%	25%	25%
**11**	0,39%	25%	6,25%
**12**	1,56%	25%	25%
**13**	0,78%	25%	25%
**14**	1,56%	25%	25%
**15**	3,12%	25%	25%
**16**	0,39%	25%	25%
**17**	0,78%	25%	6,25%
**18**	1,56%	25%	12,5%
**19**	0,78%	25%	12,5%
**20**	0,39%	25%	12,5%
**21**	1,56%	25%	12,5%
**22**	0,39%	25%	6,25%
**23**	0,78%	25%	6,25%
**24**	3,12%	25%	25%

P. aeruginosa / MBC

Strain	PHMB	LS	B
**ATCC**	1,56%	6,25%	12,5%
**1**	1,56%	6,25%	12,5%
**2**	3,13%	3,13%	12,5%
**3**	0,78%	6,25%	12,5%
**4**	3,13%	3,13%	25%
**5**	1,56%	6,25%	12,5%
**6**	3,13%	6,25%	12,5%
**7**	3,13%	12,5%	12,5%
**8**	3,13%	6,25%	12,5%
**9**	3,13%	6,25%	12,5%
**10**	0,78%	6,25%	12,5%
**11**	0,78%	6,25%	6,25%
**12**	3,13%	6,25%	12,5%
**13**	1,56%	6,25%	12,5%
**14**	3,13%	6,25%	12,5%
**15**	3,13%	12,5%	25%
**16**	3,13%	6,25%	12,5%
**17**	1,56%	6,25%	12,5%
**18**	3,13%	12,5%	12,5%
**19**	3,13%	6,25%	12,5%
**20**	0,78%	12,5%	12,5%
**21**	1,56%	6,25%	25%
**22**	1,56%	6,25%	12,5%
**23**	0,78%	6,25%	12,5%
**24**	3,13%	6,25%	12,5%

P. aeruginosa / MBEC

Strain	PHMB	LS	B
**ATCC**	12,5%	50%	50%
**1**	12,5%	50%	50%
**2**	12,5%	50%	50%
**3**	12,5%	25%	25%
**4**	12,5%	50%	50%
**5**	12,5%	50%	50%
**6**	6,25%	50%	50%
**7**	12,5%	50%	50%
**8**	12,5%	50%	50%
**9**	12,5%	50%	50%
**10**	12,5%	25%	25%
**11**	12,5%	50%	50%
**12**	12,5%	50%	50%
**13**	6,25%	50%	50%
**14**	6,25%	25%	50%
**15**	12,5%	50%	50%
**16**	12,5%	50%	50%
**17**	12,5%	50%	50%
**18**	6,25%	50%	50%
**19**	12,5%	25%	25%
**20**	6,25%	50%	50%
**21**	12,5%	50%	50%
**22**	12,5%	50%	50%
**23**	12,5%	50%	50%
**24**	12,5%	50%	50%

C. albicans / MBC

Strain	PHMB	LS	B
**ATCC**	3,12%	6,25%	12.5%
**1**	1,56%	6,25%	12.5%
**2**	1,56%	6,25%	12.5%
**3**	1,56%	6,25%	12.5%
**4**	0,78%	6,25%	12.5%
**5**	3,12%	6,25%	12.5%
**6**	0,78%	6,25%	12.5%
**7**	1,56%	1,56%	12.5%
**8**	1,56%	1,56%	6.5%
**9**	3,12%	1,56%	12.5%
**10**	0,78%	6,25%	12.5%
**11**	0,78%	3,12%	12.5%
**12**	1,56%	6,25%	6.25%
**13**	0,78%	1,56%	12.5%
**14**	3,12%	1,56%	12.5%
**15**	3,12%	6,25%	12.5%
**16**	3,12%	3,12%	12.5%
**17**	1,56%	1,56%	12.5%
**18**	0,78%	1,56%	12.5%
**19**	0,78%	6,25%	12.5%
**20**	1,56%	6,25%	12.5%
**21**	1,56%	6,25%	12.5%
**22**	0,78%	6,25%	12.5%
**23**	1,56%	6,25%	12.5%
**24**	1,56%	6,25%	12.5%

C. albicans / MBEC

Strain	PHMB	LS	B
**ATCC**	6,25%	12,5%	50%
**1**	3,12%	12,5%	50%
**2**	3,12%	6,25%	50%
**3**	3,12%	1,56%	50%
**4**	3,12%	6,25%	50%
**5**	6,25%	1,56%	50%
**6**	6,25%	12,5%	50%
**7**	6,25%	12,5%	12.5%
**8**	3,12%	12,5%	25%
**9**	25%	12,5%	50%
**10**	3,12%	12,5%	50%
**11**	3,12%	12,5%	50%
**12**	6,25%	12,5%	50%
**13**	3,12%	12,5%	12.5%
**14**	6,25%	12,5%	12.5%
**15**	6,25%	12,5%	50%
**16**	3,12%	12,5%	50%
**17**	25%	12,5%	25%
**18**	3,12%	12,5%	50%
**19**	3,12%	6,25%	50%
**20**	3,12%	1,56%	50%
**21**	25%	6,25%	25%
**22**	6,25%	12,5%	50%
**23**	6,25%	12,5%	50%
**24**	6,25%	6,25%	50%

C. acnes / MBC

Strain	PHMB	LS	B
**ATCC**	0,2%	0,4%	6,25%
**1**	0,4%	0,4%	6,25%
**2**	0,025%	0,2%	6,25%
**3**	0,025%	0,8%	6,25%
**4**	0,1%	0,8%	6,25%
**5**	0,025%	0,8%	6,25%
**6**	0,025%	0,4%	6,25%
**7**	0,1%	0,4%	6,25%
**8**	0,1%	0,8%	6,25%
**9**	0,4%	0,2%	6,25%
**10**	0,4%	0,4%	12,5%
**11**	0,2%	0,8%	6,25%
**12**	0,2%	0,8%	6,25%
**13**	0,4%	0,8%	6,25%
**14**	0,4%	0,8%	6,25%
**15**	0,025%	0,8%	6,25%
**16**	0,4%	0,8%	6,25%
**17**	0,4%	0,8%	6,25%
**18**	0,4%	0,4%	6,25%
**19**	0,4%	0,8%	6,25%
**20**	0,4%	0,8%	6,25%
**21**	0,4%	0,4%	12,5%
**22**	0,8%	0,8%	12,5%
**23**	0,4%	0,8%	6,25%
**24**	0,4%	0,8%	6,25%

C. acnes / MBEC

Strain	PHMB	LS	B
**ATCC**	3,125%	25%	50%
**1**	3,125%	25%	50%
**2**	3,125%	12,5%	50%
**3**	3,125%	25%	50%
**4**	6,25%	25%	50%
**5**	6,25%	25%	50%
**6**	3,125%	25%	50%
**7**	6,25%	25%	50%
**8**	12,5%	25%	50%
**9**	6,25%	25%	50%
**10**	6,25%	25%	50%
**11**	12,5%	50%	50%
**12**	3,125%	25%	50%
**13**	3,125%	25%	50%
**14**	6,25%	25%	50%
**15**	12,5%	25%	50%
**16**	6,25%	25%	50%
**17**	6,25%	25%	50%
**18**	6,25%	25%	50%
**19**	6,25%	50%	50%
**20**	6,25%	25%	50%
**21**	6,25%	25%	50%
**22**	6,25%	25%	50%
**23**	6,25%	25%	50%
**24**	3,125%	25%	50%

C. amycolatum / MBC

Strain	PHMB	LS	B
**ATCC**	0,4%	3,13%	50%
**1**	0,8%	3,13%	12,5%
**2**	0,8%	3,13%	12,5%
**3**	0,2%	1,56%	12,5%
**4**	0,8%	1,56%	12,5%
**5**	0,4%	1,56%	12,5%
**6**	0,2%	6,25%	25%
**7**	0,8%	1,56%	12,5%
**8**	0,8%	1,56%	50%
**9**	0,2%	6,25%	12,5%
**10**	0,8%	6,25%	12,5%
**11**	0,4%	1,56%	12,5%
**12**	0,8%	1,56%	12,5%
**13**	0,4%	3,13%	12,5%
**14**	0,8%	0,8%	12,5%
**15**	0,8%	3,13%	12,5%
**16**	0,8%	0,8%	12,5%
**17**	0,8%	3,13%	12,5%
**18**	0,8%	3,13%	12,5%
**19**	0,8%	0,8%	12,5%
**20**	0,8%	0,8%	12,5%
**21**	0,8%	3,13%	12,5%
**22**	0,8%	3,13%	12,5%
**23**	0,8%	3,13%	12,5%
**24**	0,8%	1,56%	12,5%

C. amycolatum / MBEC

Strain	PHMB	LS	B
**ATCC**	3,12%	50%	50%
**1**	12,5%	50%	50%
**2**	6,25%	50%	50%
**3**	6,25%	50%	50%
**4**	3,12%	12,5%	50%
**5**	3,12%	12,5%	50%
**6**	3,12%	50%	50%
**7**	6,25%	50%	50%
**8**	6,25%	50%	50%
**9**	6,25%	12,5%	12,5%
**10**	12,5%	50%	50%
**11**	6,25%	50%	50%
**12**	3,12%	50%	50%
**13**	3,12%	50%	50%
**14**	6,25%	50%	50%
**15**	6,25%	50%	12,5%
**16**	6,25%	50%	50%
**17**	6,25%	25%	50%
**18**	12,5%	50%	50%
**19**	6,25%	50%	25%
**20**	6,25%	50%	25%
**21**	6,25%	50%	50%
**22**	6,25%	50%	50%
**23**	12,5%	25%	50%
**24**	12,5%	25%	50%

Supplementary Material S2. The wettability of the tested solutions. H_2O – water; NaCl: saline, PHMB: 0.1% polyhexanide + poloxamer surfactant, G: low-concentrated hypochlorite; LS: 0.04% polyhexanide + macrogol surfactant; R: ringer solution; B: iodine-containing antiseptic.

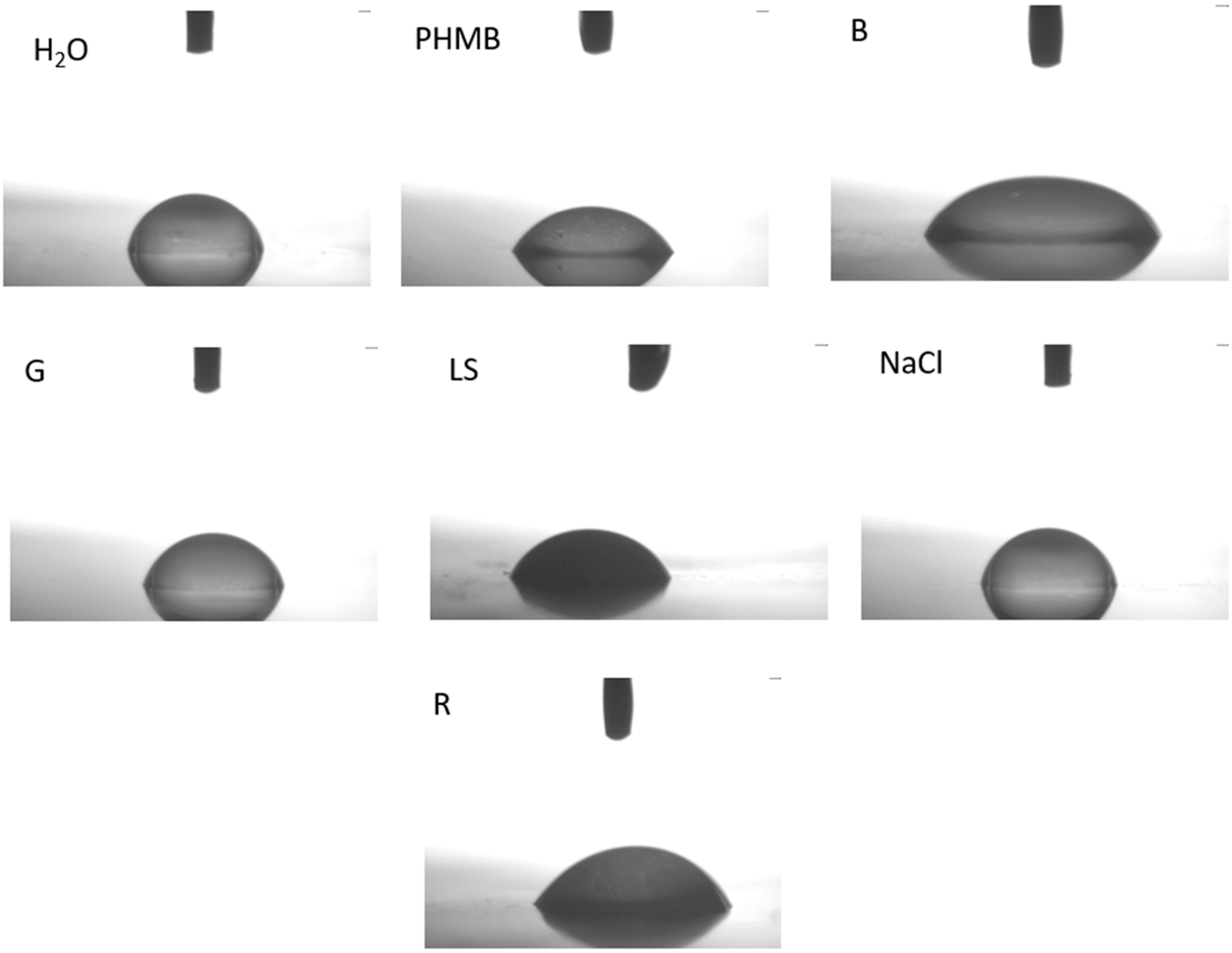

Supplement: Supplementary file 1 [file ijms-25-12720-s001.zip › ijms-3327120-supplementary.pdf]
